# Effects of a Strength and Creative Dance Intervention on Brain Electrical Activity, Heart Rate Variability, and Dual-Task Performance in Women with Fibromyalgia: A Randomized Controlled Trial Protocol

**DOI:** 10.3390/sports14020059

**Published:** 2026-02-04

**Authors:** Maria Melo-Alonso, Carmen Padilla-Moledo, Almudena Martínez-Sánchez, Lucimere Bohn, Pablo Molero, Francisco Javier Dominguez-Muñoz, Santos Villafaina, Pedro R. Olivares, Inmaculada Tornero-Quiñones, Juan Luis Leon-Llamas, Narcis Gusi

**Affiliations:** 1 Grupo de Investigación en Actividad Física, Didáctica de la Expresión Musical, Plástica y Corporal, Facultad de ciencias del Deporte, Calidad de Vida y Salud (AFYCAV), Universidad de Extremadura, Avenida de la Universidad s/n, 10003 Cáceres, Spain; mmeloa@unex.es (M.M.-A.); pmolero@unex.es (P.M.); fjdominguez@unex.es (F.J.D.-M.); svillafaina@unex.es (S.V.); ngusi@unex.es (N.G.); 2 Instituto de Investigación e Innovación en Deporte (INIDE), 10003 Cáceres, Spain; 3 GALENO Research Group, Department of Physical Education, Faculty of Education Sciences, University of Cadiz, 11519 Puerto Real, Spain; carmen.padilla@uca.es; 4 Instituto de Investigación e Innovación Biomédica de Cádiz (INiBICA),11009 Cádiz, Spain; 5 Research Center in Physical Activity, Health and Leisure (CIAFEL), Faculty of Sports, University of Porto (FADEUP), 4200-450 Porto, Portugal; lucimerebohn@fade.up.pt; 6 Laboratory for Integrative and Translational Research in Population Health (ITR), University of Porto, 4050-600 Porto, Portugal; 7 Facultad de Educación, Psicología y Ciencias del Deporte, Universidad de Huelva, 21007 Huelva, Spain; pedro.olivares@ddi.uhu.es (P.R.O.); inmaculada.tornero@dempc.uhu.es (I.T.-Q.); 8 Biomedical Research Networking Center on Frailty and Healthy Aging (CIBERFES), 45071 Toledo, Spain

**Keywords:** activities of daily living, creative, EEG, exercise, fall, stimulus–response paradigm

## Abstract

Fibromyalgia is a complex chronic disorder involving persistent widespread pain accompanied by functional limitations, cognitive impairments, and alterations in neural processing. Previous research indicates that exercise-based interventions can play a key role in alleviating symptom burden and enhancing physical performance; however, there is limited evidence regarding their impact on neurophysiological mechanisms. Creative dance, in combination with strength training, may stimulate both motor and cognitive systems, promoting brain plasticity and functional improvements. This study will analyze the effects of a six-week strength and creative dance program on physical fitness under single- and dual-task conditions in women with fibromyalgia and will explore the associated changes in brain electrical activity and autonomic modulation. Methods: This randomized controlled trial will be divided into an exercise group (*n* = 22) and a control group (*n* = 22). The 6-week supervised intervention consists of two 60-min sessions per week, combining strength exercises and creative dance. Primary outcomes include physical fitness tests (strength, mobility, balance, and agility gait test in single-task and dual-task), fibromyalgia symptoms, and quality of life. Secondary outcomes include changes in electroencephalography, heart rate variability, physical activity level, and fear of falling. Statistical analyses will compare within- and between-group differences using non-parametric tests and effect sizes. It is hypothesized that the intervention will improve physical fitness and dual-task performance, alongside increases in brain activity power. This study may provide insights into the neurophysiological mechanisms underlying the benefits of exercise benefits in fibromyalgia.

## 1. Introduction

Fibromyalgia (FM) is a long-term rheumatic condition of unclear etiology that predominantly affects women. It is characterized by widespread musculoskeletal pain as well as reduced physical capacity, balance impairments, fatigue, depression, anxiety, and sleep disturbances [1]. Individuals with FM who present a high risk or fear of falling [2] tend to reduce their motor activities, generating a cycle of physical deterioration, decreased quality of life, loss of autonomy, and a greater degree of disability [3].

Physical exercise has been shown to reduce pain [4], symptom severity, and fatigue [5] as well as to improve quality of life, physical fitness, sleep [5], and psychological well-being [5]. Multicomponent programs that combine aerobic and strength exercises are particularly effective for decreasing pain and enhancing both physical and mental health. Other modalities, such as whole-body vibration [6], aquatic exercise [7] or virtual reality-based interventions, have also demonstrated benefits in functional capacity, along with a reduction in both the risk and fear of falling.

Among the most recent interventions, dance-based therapies have emerged as a promising alternative capable of improving pain, quality of life, depressive symptoms, and the overall impact of the disease [8]. Two main approaches can be distinguished: (a) repetitive dance, focused on performing structured movement sequences such as aerobic dance or Zumba, and (b) creative or improvised dance, based on the free expression of bodily movement, as in biodance, aquatic dance, or dance movement therapy. Both types of dance have been shown to improve overall physical condition and reduce pain [9] as well as mitigate the impact of disease and contribute to improvements in health-related quality of life, although some studies suggest that creative dance may be more effective [8]. From a physical perspective, dance promotes balance, coordination, strength, flexibility, aerobic capacity, and proprioception [10]. At the cognitive level, it enhances attention, memory, and decision making [10], while in the psychosocial domain, it fosters motivation, emotional expression, and social interaction [11]. Altogether, dance constitutes a comprehensive intervention that simultaneously stimulates the motor, sensory, and cognitive systems, producing positive effects on mood, daily functioning, and social life. Therefore, dance supports brain plasticity [12] and contributes to overall improvements in function and quality of life in individuals with FM.

Studies in older adults indicate that dance-based interventions are associated with structural and functional brain changes in regions involved in executive function, attention, and cognitive control as well as with increased levels of brain-derived neurotrophic factor, supporting their role in neuroplasticity [10]. In individuals with fibromyalgia, exercise-based interventions have been shown to modulate brain electrical activity, including changes in beta band [13], which may contribute to improvements in cognitive performance and the normalization of altered neural oscillations associated with pain [14] and cognitive dysfunction [15].

Beyond static neurophysiological measures, the integration of perception and action represents a relevant framework for understanding exercise-related adaptations in real-life contexts. Dual-task paradigms, which reflect everyday functional demands, may be particularly sensitive to changes induced by dance-based interventions. The theoretical interpretation of these mechanisms, including their relation to stimulus–response integration models such as the Binding and Retrieval in Action Control (BRAC) framework, is further addressed in the expected mechanisms section.

Previous research supports the feasibility and effectiveness of relatively short-duration (six weeks) exercise interventions in FM. In this regard, Casanova-Rodríguez et al. [16] reported that statistically significant differences can be observed in aerobic exercise programs lasting from three to twenty-four weeks, and this duration range has also been applied in strength training interventions [17]. Taken together, these findings suggest that a six-week intervention period is enough to produce clinically meaningful changes and is supported by evidence in the FM literature.

However, despite growing evidence supporting the benefits of creative dance-based and multicomponent exercise interventions in fibromyalgia, no previous studies have simultaneously examined their effects on physical performance under single- and dual-task conditions together with resting-state EEG markers of neuroplasticity, particularly alpha and theta activity. Overall, these findings support the use of a multidimensional intervention combining strength training and creative dance to target both physical and cognitive impairments in individuals with FM, forming the basis for the purposes of the present study.

Objectives:

(a) To analyze the effects of a 6-week physical exercise training program based on creative dance combined with strength training on physical fitness (strength, mobility, balance, functional capacity, and gait agility) under both single- and dual-task conditions in individuals with FM;
(b) To investigate exercise-related changes in resting-state brain electrical activity and HRV as a secondary outcome, with a primary focus on beta power, in order to explore potential neurophysiological mechanisms underlying functional improvements.

Hypotheses:

**H1.** Six weeks of strength training and creative dance are expected to significantly improve physical fitness (strength, mobility, balance, functional capacity, and gait agility) in individuals with FM, under both single- and dual-task conditions, compared with their baseline status and a control group.
**H2.** It is hypothesized, for exploratory purposes, that the physical exercise intervention will be associated with specific changes in resting-state brain electrical activity, characterized primarily by increased beta power, that could be related to increased cerebral blood flow [13]. Also, the autonomic modulation would be enhanced [18].

## 2. Materials and Methods

### 2.1. Study Design

This study will be a randomized controlled trial with a parallel group design (pretest–post-test control group design) [19]. Participants will be recruited from FM associations in the region of Extremadura (Cáceres, Almendralejo, Hervás and Olivenza) in Spain. Each FM association will be randomly assigned to either the intervention group, which will participate in a structured physical activity program or to the control group. To ensure methodological rigor and high-quality reporting consistency with clinical trial standards, the study will follow the SPIRIT 2013 Statement guidelines and will apply the TESTEX 2015 scale, a tool specifically developed to evaluate the quality and reporting standards of exercise training studies [20].

### 2.2. Participants

#### 2.2.1. Eligibility Criteria

To be eligible for participation, individuals must meet the following inclusion criteria: (a) women with a clinical diagnosis of FM provided by a rheumatologist according to the American College of Rheumatology criteria [21]; (b) ability to ambulate independently without assistance; (c) be able to communicate with the research staff; and (d) have read, understood, and signed the informed consent form. The exclusion criteria will be (a) the presence of psychiatric or neurological conditions; (b) current or past substance abuse or dependence; (c) contraindication for physical exertion; (d) severe fractures or musculoskeletal injuries within the previous six months; (e) being pregnant; (f) active or ongoing ear diseases or infections that may affect balance; and (g) any other condition that could impair balance.

#### 2.2.2. Recruitment

The sample size will be calculated based on one of the study’s primary outcomes, i.e., the impact of FM. Data from a previous study [22] that will be used as a reference evaluated the effects of a creative dance intervention on this outcome. In that study, the impact of FM was reduced by 1.16 points compared with the control group after six weeks of intervention.

According to the results obtained in the G*Power statistical power analysis software version 3.1.9.7 (Heinrich Heine University Düsseldorf, Düsseldorf, Germany), assuming an effect size of f(V) = 0.98, a power of 85%, and a significance level of 0.05, a sample size of 22 people per intervention group is estimated. However, considering an anticipated dropout rate of 10%, a total of 44 participants (22 per group) will be needed to observe a significant effect.

Therefore, the participants recruitment will take place between January and March 2026. Both groups will consist of women with FM (exercise group: *n* = 22; and control group: *n* = 22) recruited from different FM associations in the region of Extremadura.

### 2.3. Randomization of Assessments

The assessments will follow a fixed sequence with alternating tests of the upper and lower limbs, thereby ensuring adequate rest periods to prevent cumulative fatigue and optimizing the overall time required for the evaluations. To reduce familiarization and learning effects, the sequence of specific conditions will be randomized. Randomization will apply to both the physical test conditions and the arithmetic cognitive task.

The physical tasks that each participant will complete at baseline (pre) and post-intervention will be scheduled across two assessment days:

- Day 1: Cognitive task (arithmetic-based counting test), handgrip strength, timed up and go test (TUG), arm curl test (ACT), and 30 s chair stand test (CST). Randomization: The random allocation sequence will be generated by an independent researcher using a computer-based random number generator (Random Number Generator tool; Google, LLC., Mountain View, CA, USA) and implemented via a password-protected Excel file. Simple randomization (0/1) will be used for Day 1 tasks. Personnel responsible for enrolling participants and conducting assessments will remain blinded to the random allocation sequence. Two aspects will be randomized for each participant: (1) whether the arithmetic subtraction task is performed at the beginning (coded as 1) or at the end (coded as 0) of the physical test sequence and (2) whether the motor task is performed as a single-task condition (coded as 0) or as a dual-task combined with the cognitive subtraction task (coded as 1). The handgrip test will be performed without dual-task condition due to its temporal execution characteristics;
- Day 2: Baseline resting Electroencephalography (EEG) and Heart Rate Variability (HRV), EEG and HRV combined with the Stimulus–Response (S-R) paradigm protocol, Short Physical Performance Battery (SPPB), and Agility Challenge for the Elderly (ACE). Randomization: Only the spatial orientation component of the ACE test will be randomized according to the recommendations of Lichtenstein et al. [23]. The random allocation sequence for this task will also be generated by the independent researcher, recorded in the same password-protected Excel file, and accessed by the researcher immediately prior to testing to ensure allocation concealment

### 2.4. Clusters Randomization

The clusters in this study are FM associations from the region of Extremadura (Cáceres, Almendralejo, Hervás, and Olivenza) in Spain. Each association will be randomly assigned to either the intervention group, which will participate in a structured physical activity program, or to the control group. The allocation sequence for the associations will be created using a computer-generated random number system (Random Number Generator tool; Google, LLC., Mountain View, CA, USA). Simple randomization will be applied, with no stratification, given the small number of clusters. The sequence will be recorded in a password-protected Excel file. Personnel responsible for enrolling associations and implementing the intervention will remain blinded to the allocation sequence until each cluster is assigned. The principal investigator (IP) will provide the group assignment immediately prior to the start of the intervention for each association, ensuring allocation concealment.

### 2.5. Blinding

Participants and personnel responsible for delivering the physical activity intervention will not be blinded due to the nature of the intervention, as they will become aware of their group allocation. However, the data analysts and evaluators will be blinded to group allocation to minimize bias in assessment and analysis bias. Group assignments will be coded as A or B in all data collection forms and databases, without revealing which code corresponds to the intervention or control group.

Unblinding will only be permissible in exceptional circumstances, such as a medical emergency requiring knowledge of the participant’s assigned group. In such cases, the IP for generating the allocation sequence will reveal the assignment following a documented procedure, and the reason for unblinding will be recorded.

### 2.6. Ethical Approval

The study protocol was approved by the Research Ethics Committee of the University of Extremadura (approval reference: 55/2021). The trial was registered in the Open Science Framework (OSF) under the title Cost-effectiveness of Autonomous Smart Insole–based physical exercise on brain, motor pattern and quality of life in FM (AUSIEX) (registration identifier: https://doi.org/10.17605/OSF.IO/Q6GZ4 on 3 June 2025. Participants will receive a detailed explanation of the procedures and will be provided written informed consent, following the principles outlined in the Declaration of Helsinki.

### 2.7. Procedures

**Figure 1:**
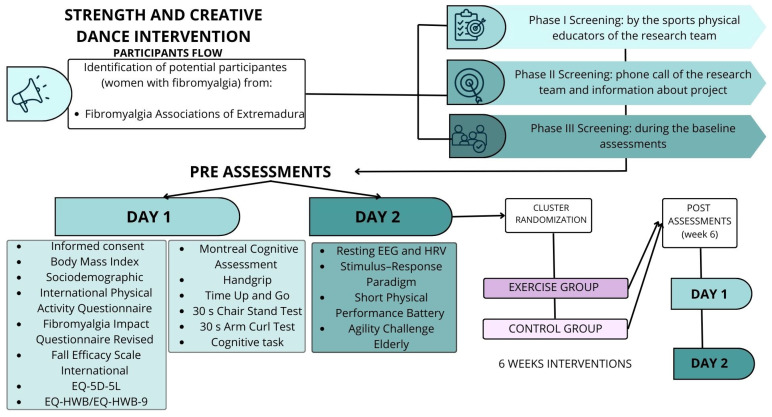
Timeline of the study.

Figure 1 illustrates the study timeline and the sequence of the interventions. Initially, anthropometric measurements will be obtained to calculate participants’ body mass index (BMI). Subsequently, participants will provide sociodemographic information and will complete the following questionnaires: the International Physical Activity Questionnaire (IPAQ); the FM Impact Questionnaire-Revised (FIQ-R); the Falls of Efficacy Scale International (FES-I); the EuroQol 5 dimensions-5 levels (EQ-5D-5L); and the EQ-Health and Wellbeing (EQ-HWB and EQ-HWB-9).

Pre-intervention and post-intervention assessments: On the first day, after completing the questionnaires, participants will undergo a cognitive assessment using the Montreal Cognitive Assessment (MoCA). This will be followed by the physical (strength and mobility test) and cognitive task, which will be administered under both single-task and dual-task conditions. On the second day, the following assessments will be conducted: (1) a 5-min baseline resting EEG, (2) Stimulus–Response (S-R) paradigm, and (3) physical performance tasks, including balance and agility gait tests.

All physical performance and outcome assessments will be conducted by evaluators blinded to participants’ group allocation, as described in Section 2.4.

### 2.8. Intervention

Participants in the exercise group will take part in a six-week physical exercise training program, while those in the control group will continue their usual daily routines. The exercise group will be submitted to an intervention consisting of two group-based sessions per week, lasting 60 min, with a maximum of ten participants per group. Sessions will be held in facilities provided by the collaborating FM associations or University of Extremadura. The program will focus on balance, postural control, coordination, mobility, aerobic conditioning, and strengthening of both upper and lower limbs, with particular emphasis on the practice and learning compensatory stepping strategies [24] through creative movement. The proposed exercises are specifically designed to encourage adaptive stepping responses to balance challenges, promoting effective motor strategies to regain stability under dynamic and potentially destabilizing situations. All sessions will be supervised by a qualified physical exercise professional with a degree in Physical Activity and Sport Sciences and a Master’s degree in Health. The trainer will have at least five years of experience working with special groups, specifically individuals with FM, and will provide continuous technical feedback to ensure the correct execution of movements.

Each session will be divided into two parts of 30 min. The first part will include the strength training exercise and the second the creative dance training, based on the fundamental components of creative dance [25]. A typical session will include five to ten min of warm-up exercise, followed by a series of strength exercises designed to enhance muscular resistance and a creative dance training phase focused on motor problem-solving tasks.

- The strength training will include exercises for both the upper (biceps curls, triceps extensions, rows, shoulder presses, chest presses, and lateral and front raises) and lower body (squats and their variations, deadlifts, forward and backward lunges, and hip thrusts) as well as abdominal work (crunches, Russian twists, oblique exercises, dead bugs, isometric and dynamic planks, and Pallof presses). Training volume will be regulated through the number of sets and repetitions, and intensity will be progressively increased by adjusting exercise difficulty. Exercises will primarily be performed using body weight, with elastic resistance bands (~5 kg resistance) incorporated intermittently as supplementary equipment. All sessions will be accompanied by background music (see Supplementary Material Table S1);
- The creative dance training: In this study, creative dance is defined as a structured movement-based intervention in which participants engage in guided improvisation, expressive movement, and continuous decision-making in response to internal and external cues. Essential components include (1) improvisation within predefined movement constraints, (2) active selection among multiple motor solutions, (3) perception–action coupling to respond to dynamic stimuli, and (4) expressive and exploratory movement rather than repetitive patterns. Creativity in the sessions is systematically induced through specific task instructions and variations, aiming to enhance motor adaptability and cognitive flexibility. The creative dance will consist of five main exercises, each with its corresponding variations. Two of them (Trajectories and Passing) will follow a fixed structural pattern while being adapted according to the daily objective and the specific element of creative dance being addressed. Two sessions will be dedicated to each dance component: body, weight, contact, space, time, and interaction [26]. Exercise intensity will be monitored through movement speed and task duration. All sessions will be accompanied by either background or directive music (see Supplementary Material Table S1). Supplementary Material Document S1 contains the detailed of information about the exercise and the entire exercise program itself.

### 2.9. Control of Training Intensity

After each training session, participants will complete the Visual Analog Scale (VAS), the Borg Rating of Perceived Exertion (Borg-RPE), and the Borg Category-Ratio 10 scale (Borg-CR10) questionnaires to evaluate perceived pain and exertion.

**Figure 2:**
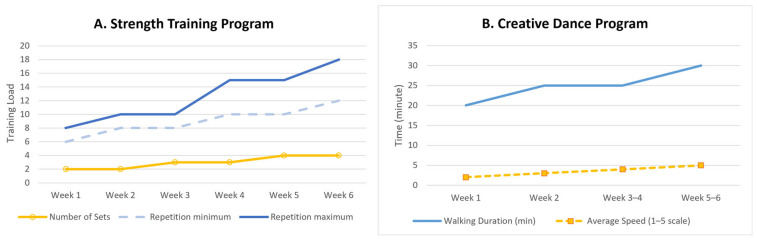
Progressive Load Evolution in Both Training Modalities. Graph (**A**) (**left**): Progressive increase in sets and repetitions across the six weeks of the strength training program. Graph (**B**) (**right**): Increment in total walking duration and mean walking.

The strength training program will adhere to the training principles established by the American College of Sports Medicine (ACSM) and will employ a linear progressive model [27]. Training volume and repetitions will progressively increase across the six weeks; see Figure 2A:

- Week 1: Two sets of 6–8 repetitions;
- Week 2: Two sets of 8–10 repetitions;
- Week 3: Three sets of 8–10 repetitions;
- Week 4: Three sets of 10–15 repetitions;
- Week 5: Four sets of 10–15 repetitions;
- Week 6: Four sets of 12–18 repetitions.

Participants will be instructed to work within these ranges according to their physical condition and pain levels. Those unable to reach the prescribed repetitions due to FM symptoms or physical limitations will be encouraged to perform as many repetitions as possible with the aim of gradual improvement. Conversely, participants feeling well and without symptom flare-ups will be invited to perform up to 5–8 additional repetitions beyond the prescribed range.

The creative dance training program includes creative motor tasks, such as free or improvised movement, that are designed to promote multiple and original motor responses. Similar to the strength training program, it will follow ACSM guidelines and will adopt a linear progressive model [27]. Training intensity will be primarily regulated through walking speed, while spatial variation (i.e., reducing or expanding the movement area) will serve as an additional method to increase or decrease exercise difficulty (see Figure 2B).

Participants will receive a reference scale for walking speeds to be used throughout the intervention: 0 = stop; 1 = slow; 2 = comfortable walk; 3 = brisk walk; 4 = fast walk; and 5 = very fast walk.

- Week 1: Mostly stationary exercises and 20 min of walking at an average speed of level 2;
- Week 2: A total of 20 min of walking at an average speed of level 3, and 10 min of semi-static activities;
- Week 3–4: A total of 20 min of walking at speeds between levels 3–5, plus 10 min of semi-static work;
- Week 5–6: A total of 30 min of walking at speeds between levels 3–5.

### 2.10. Adherence, Attendance, and Compliance

Adherence will be evaluated based on attendance and compliance. Attendance will be defined as the percentage of sessions attended by each participant, as recorded by the physical exercise trainer, relative to the total number of scheduled sessions over six weeks, with two sessions per week, totaling 12 sessions. Minor variations in the total number of sessions may occur due to holidays or logistical reasons during intervention phases or for individual participants. Compliance will be defined as the percentage of sessions completed in accordance with the intervention protocol, calculated as the number of sessions completed according to protocol divided by the number of sessions with valid data, expressed as a percentage. A session will be considered completed when the participant:(1) performs the full 60-min training session (30 min of strength training and 30 min of creative dance training) and (2) completes the three post-session questionnaires assessing pain and perceived exertion (both the short and long versions of the Borg Scale).

### 2.11. Safety and Adverse Events

Participant safety will be monitored continuously throughout the program by the physical exercise professionals, who will deliver the intervention. He/she will record any adverse events occurring during or immediately after each session. An adverse event will be defined as any unexpected medical occurrence, symptom, or injury arising during participation, regardless of whether it is deemed related to the intervention. Given the nature of the program, potential minor adverse events may include transient muscle soreness or fatigue.

### 2.12. Outcome

#### 2.12.1. Primary Outcomes Measures (Confirmatory Analyses)

(A) Questionnaires

Impact of the Disease

The FM Impact Questionnaire Revised (FIQR) [28] is a self-administered instrument composed of 21 items. Each item is rated on a 0–10 scale, with higher scores indicating greater disease impact. The questionnaire is structured into three domains: (a) function, (b) overall impact, and (c) symptoms. The total score, obtained by summing the three domains, ranges from 0 to 100, with higher values reflecting a greater overall impact of FM. In this study, the validated Spanish version of the FIQR [29] will be applied.

Health-Related Quality of Life

The EuroQol-5 dimensions 5 levels (EQ-5D-5L) measures health-related quality of life across five domains (mobility, self-care, usual activities, pain/discomfort, and anxiety/depression), each with five response levels. It also includes a visual analog scale ranging from 0 (worst health) to 100 (best health). The instrument has been validated in Spanish populations [30].

(B) Physical Fitness

Handgrip Strength

The Handgrip Strength (HGS) test will be used to assess maximal isometric hand strength. Participants will stand upright with their elbow extended at hip height and exert maximal force on a hand-held dynamometer (Takei TKK 5401, Takei Scientific Instruments Co., Ltd., Tokyo, Japan) featuring analog grip and adjustable handle to accommodate various hand sizes. Two repetitions will be performed for each hand, with a one-min rest interval between attempts. The highest value from each hand will be used for analysis [31].

Timed Up and Go

The Timed Up and Go (TUG) test is a dynamic test of mobility and balance in which participants rise from a chair without using their hands, walk 3 m as quickly as possible without running, turn around a cone, return to the chair, and sit down [32]. Performance is measured as the time taken to complete the task with ChronoJump software (version 2.2.0 Barcelona, Spain) and Chronopic hardware (DIN A-4, 210 × 297 mm), which will be placed on the backrest of the chair; one practice trial will be allowed for familiarization, after which the test will begin with the command “Ready, go”.

Short Physical Performance Battery

The Short Physical Performance Battery (SPPB) evaluates lower-body functional capacity through three components: tasks assessing standing balance, usual gait over 4 m, and the five-repetition sit to stand test [33]. Each component is scored from 0 to 4, with lower scores indicating greater risk of frailty, disability, and falls. The total score ranges from 0 to 12 and is categorized as dependent (0–3), frail (4–6), pre-frail (7–9), and robust (10–12) [33].

30 s Chair Stand Test

The 30 s Chair Stand Test (CTS) will be used to assess lower-limb strength and power. Participants will sit with their arms crossed at chest level, stand to full knee extension, and return to the starting position until the backrest is touched, repeating the cycle as many times as possible in 30 s [34]. Two practice trials will be allowed for familiarization, after which the test will begin with the command “Ready, go”. Performance will be recorded using the ChronoJump software (version 2.2.0, Barcelona, Spain) and Chronopic hardware (DIN A-4, 210 × 297 mm, ChronoJump, Barcelona, Spain), which will be placed on the seat of the chair. Only correctly executed repetitions will be counted. This test has shown good reliability and validity in women with FM [35].

30 s Arm Curl Test

The 30 s Arm Curl (ACT) test will be used to assess upper-limb strength and endurance. Participants will sit in a chair with their feet flat on the floor and their back supported. Grasping a 2.5 kg weight in the dominant hand while maintaining a neutral wrist position, they will perform repeated elbow flexion and extension through the full range of motion for 30 s [34]. Two practice attempts will be allowed for familiarization, after which the test will begin with the command “Ready, go”. The total number of correctly executed repetitions will be recorded. This test has demonstrated good reliability in women with FM.

Agility test

The Agility Challenge for the Elderly (ACE) test will be used to assess performance under fall-threatening, real-life-like motor demands. The ACE consists of a standardized course set on a volleyball court, comprising three domains: stop and go, cutting maneuvers, and spatial orientation, each targeting a specific aspect of agility. Participants will complete four trials per session, interspersed with at least 3 min of rest; the first attempt on each testing day will serve as a familiarization trial. Instructions are to walk as fast as possible without running. Errors in the execution of the spatial orientation task (incorrect order) will be recorded. To avoid learning effects, cone numbers will be individually randomized for each trial. The ACE has demonstrated high reliability and validity in older adults [23].

Dual-Task Condition

Under dual-task conditions, participants will be required to execute a physical task (TUG, ACT, and CST) while concurrently engaging in a cognitive activity. The cognitive task will involve performing serial subtractions by two, starting from a randomly selected odd number greater than 100.

#### 2.12.2. Secondary Outcomes Measures (Exploratory Analyses)

(A) Questionaries

Sociodemographic Information

Participants will report demographic information (level of education and occupation) and clinical information (age, current medications, diagnosed conditions, history of falls, pain level measured by a visual analog scale, injuries, and other relevant health data).

Anthropometric Measurement

Body composition will be assessed using the Tanita Body Composition Analyzer BC-418 MA (Tanita Corp., Tokyo, Japan) along with a SECA 769 column scale and stadiometer (SECA Corp., Hanover, MD, USA).

Physical Activity Level

The International Physical Activity Questionnaire (IPAQ) Short Version will be used to assess physical activity and sitting time. This has been used to calculate total metabolic equivalents (METs) per week and the amount of time spent sitting [36]. The short Spanish version of the IPAQ will be used in this study and has shown strong reliability and validity among in Spanish populations [37].

Fear of Falling

The Fall Efficacy Scale International (FES-I) is a self-report questionnaire consisting of 16 items, with each rated on a four-point scale. The higher the score, the greater the fear of falling. The Spanish version of FES-I will be used [38], which has shown high reliability and validity [39].

Health and Wellbeing

The EQ-Health and Wellbeing (EQ-HWB) will be used to evaluate health and wellbeing in the study population [40]. This generic instrument, developed by the EuroQol Research Foundation, includes a 25-item version that assesses multiple domains such as activity, relationships, cognition, self-identity, autonomy, emotional state, and physical sensations. A shorter version, the EQ-HWB-9 (nine items), will also be administered, focusing on core aspects including mobility, daily activities, loneliness, concentration/clarity of thought, sadness/depression, anxiety, pain, and fatigue. The Spanish versions of the EQ-HWB and EQ-HWB-9 [41], which have demonstrated good validity, will be administered It should be noted that the EQ-HWB and EQ-HWB-S are currently classified as experimental instruments under the Intellectual Property Policy of the EuroQol Research Foundation.

Cognitive Impairment

Montreal Cognitive Assessment (MoCA) is a short screening instrument intended to assess various cognitive domains, such as attention, executive functions, memory, language, visuospatial abilities, calculation, and orientation [42]. In this study, a cut-off score of 23/30 was applied to enhance diagnostic accuracy and minimize false positives [43].

Perceived Exertion

The Borg Rating of Perceived Exertion (Borg-RPE) scale is a tool used to monitor exercise intensity. The original RPE scale ranges from 6 (“no exertion at all”) to 20 (“maximal exertion”) [44]. The modified version, the Borg Category-Ratio 10 scale (Borg-CR10), ranges from 0 (“nothing at all”) to 10 (“extremely strong”) [45]. Both scales have demonstrated high reliability and validity [46]. The Spanish versions of both scales will be administered.

(B) Neurophysiological Variables

Electroencephalography and Heart Rate Variability

Participants will be seated in a quiet room with their feet flat on the floor and eyes open and instructed to remain quiet for five min to acquire baseline resting Electroencephalography (EEG) and Heart Rate Variability (HRV) data. During this procedure, an adhesive electrode (Kendall™ H1224SG, CardinalHealth™, Dublin, OH, USA) will be placed in the left lower thoracic region, below the breast area, over the intercostal space corresponding to the cardiac region, to record cardiac activity. EEG and HRV will be recorded using the Enobio^®^ system instrument (Neuroelectrics, Cambridge, MA, USA) with the NIC2 software, version 2.1.4.1 (for Windows) [47]. This equipment has demonstrated high reliability, even with dry electrodes [48]. EEG activity will be obtained from 19 channels following the international 10–20 system, covering frontal (Fz, Fp1, Fp2, F3, F4, F7, and F8), central (Cz, C3, and C4), temporal (T3, T4, T5, and T6), parietal (Pz, P3, and P4), and occipital (O1 and O2) areas, using the mastoids as references. Impedance will be maintained below 10 kΩ. Signals will be sampled at 500 Hz, applying a 50 Hz notch and a 1–40 Hz bandpass filter. For data processing during movement tasks, recommendations from Cheron et al. [49] will be followed, applying an Artifact Subspace Reconstruction (ASR) filter [50]. Preprocessing and analysis will be performed in EEGlab (v2021, Matlab toolbox; San Diego, CA, USA), where artifacts from ocular, muscular, or electrical sources will be removed using Independent Component Analysis (ICA) [51]. Cleaned data will then be divided into spectral bands: theta (4–7 Hz), alpha-1 (8–10 Hz), alpha-2 (11–12 Hz), beta-1 (13–18 Hz), beta-2 (19–21 Hz), and beta-3 (22–30 Hz) using the pop_spectopo.m function.

HRV signals will be exported in .edf format and analyzed with Kubios HRV software (version 2.1) [52]. The extracted metrics will include: (a) time-domain variables—mean HR, RR, SDNN, Pnn50, and rMSSD; (b) frequency-domain indices—low frequency (LF)/high frequency (HF) ratio (LF/HF) and total power; and (c) nonlinear parameters—approximate (ApEn) and sample entropy (SampEn).

Stimulus–Response Paradigm

Stimulus–Response (S-R) paradigm, a modified version of the methodology proposed by Colzato et al. [53] and Rawish et al. [54], will be employed and adapted to better approximate real-life contexts. Whereas the original studies examined the neural processes involved in file event processing, our approach will incorporate scenarios with greater ecological validity.

Participants will be seated in front of a 15.6-inch (39.6 cm) computer screen, maintaining a viewing distance of approximately 60 cm. The stimulus–response paradigm will be implemented using OpenSesame software, version 4.0.29 (for Windows, OpenSesame Inc., Portland, OR, USA), developed by Sebastiaan Mathôt: https://osdoc.cogsci.nl/4.0/download/ (accessed on 3 September 2024), for the presentation of visual stimuli. The paradigm will follow the sequence described below (see Figure 2):

- Pre-Cue: A black screen with a white plus symbol (“+”, 64 × 64 pixels) will be displayed for 1500 milliseconds;
- Cue: A 1500-millisecond video will be shown featuring a woman walking either to the left or right across a pedestrian crossing. The walking direction will be randomized;
- Pre-S1: An image of a store logo will be presented for 1000 milliseconds;
- Stimulus S1: A second video (3000 milliseconds) will be shown, in which the same woman will continues walking on the pedestrian crossing and displays an unexpected behavior in response to a balance loss. To recover, she performs a compensatory stepping strategy: either a lateral step or a forward step. A visual distractor (a red or grey car) will also be present in the background;
- Pre-S2: The same store logo image will be shown again, now for 2000 milliseconds;
- Stimulus S2: A second instance of the stimulus will be presented, with the same characteristics as S1 but randomized.

The paradigm will include two conditions related to feature overlap between S1 and S2:

- Complete feature overlap (S1 = S2): Same stepping strategy and same car color;
- No feature overlap (S1 ≠ S2): Different strategy and different car color.

During each trial (S1 and S2), participants will provide two responses (R1 and R2) by pressing either the left or right control key with their index fingers.

- R1: Participants will memorize the walking direction shown in the Cue phase (left or right) and respond with the corresponding key when S1 appears;
- R2: Participants will quickly indicate the stepping strategy shown in S2: the left key for a forward step and the right key for a lateral step.

This task structure will allow for the identification of four conditions based on response and feature overlap:

(a) Stimulus feature overlap with response repetition (S1 = S2 and R1 = R2);
(b) Stimulus feature overlap with response alternation (S1 = S2 and R1 ≠ R2);
(c) No stimulus feature overlap with response repetition (S1 ≠ S2 and R1 = R2);
(d) No stimulus feature overlap with response alternation (S1 ≠ S2 and R1 ≠ R2).

The full task consists of 80 trials, distributed across five blocks of 40 trials each. Reaction times and response accuracy will be recorded throughout the experiment. Prior to the actual task, participants will complete a 5-min practice session without wearing the EEG recording cap.

**Figure 3:**
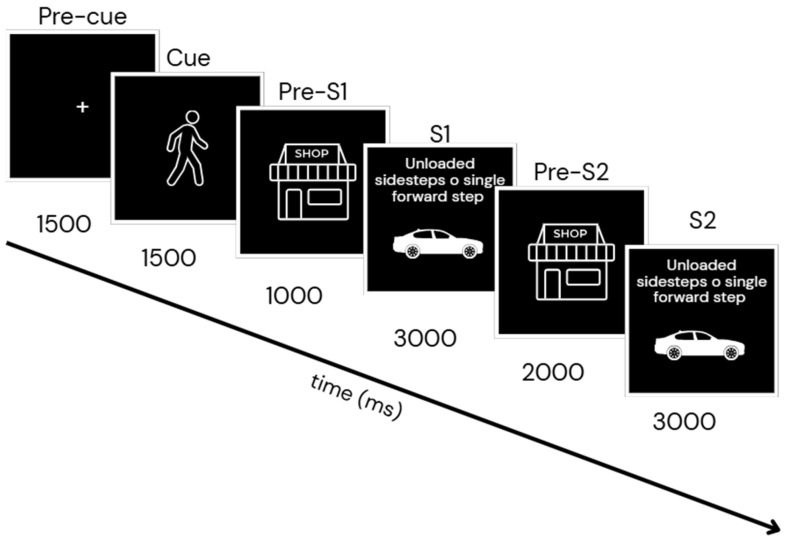
Graphical representation of a single trial within the stimulus–response (S-R) paradigm adapted from Colzato et al. [53]. The temporal structure of the task is illustrated along a timeline. The pre-cue phase corresponds to the interval preceding cue presentation (from −9000 to −7500 ms relative to S2). The pre-S1 phase spans from to 6000 to 5000 ms, followed by the post-S1, which extends from 5000 to 2000 ms before the onset of S2. The pre-S2 interval covers the 2000 ms immediately preceding S2 (prone), whereas the post-S2 interval corresponding to the within-trial phase includes the 1000 ms following probe onset. Participants will be instructed to memorize the direction indicated by the cue. When stimulus S1 appears, they will respond by indicating the cue direction using the left or right control key. Upon presentation of S2, participants will identify the required movement, selecting the right control key for unloaded sidesteps and the left control key for a single forward step.

During this test, EEG and HRV will be recorded. To achieve this, the Enobio^®^ instrument (Neuroelectrics, Cambridge, MA, USA) and the OpenSesame software, version 4.0.29 (for Windows, OpenSesame Inc., Portland, OR, USA)will be synchronized using the Lab Streaming Layer (LSL). EEG time–frequency analysis will be performed specifically within this paradigm to examine neural dynamics associated with the different task events. EEG data will be epoched relative to the onset of S2 (0 ms) and segmented according to task phases (Figure 3): Pre-Cue (−9000 to −7500 ms), Pre-S1 (−6000 to −5000 ms), S1 (−5000 to −2000 ms), Pre-S2 (−2000 to 0 ms), and the within-trial interval (0 to 1000 ms). Time–frequency decomposition will be conducted using Morlet wavelets (width = 5 cycles) with a Hanning taper. For each electrode and time point, the average spectral power will be computed within the theta (4–7 Hz), alpha (8–12 Hz), and beta (12–30 Hz) frequency bands. Comparisons will be performed between stepping strategies (lateral vs. forward) and task load (single-task vs. dual-task), with pointwise *t*-tests calculated for each time point in the 0–1000 ms window after S2 onset. False-Discovery Rate (FDR) correction will be applied to account for multiple comparisons, and effect sizes (r) will be reported for significant differences.

### 2.13. Data Analysis

The collected outcomes will be compiled in an anonymized database for statistical processing. Both descriptive statistics and inferential analyses will be conducted. Data analysis will be performed using the Statistical Package for the Social Sciences software (SPSS, version 25.0; IBM Corp., Armonk, NY, USA). The Shapiro–Wilk and Kolmogorov–Smirnov test will be used to assess the normality of all variables.

Furthermore, to explore the effect of physical exercise intervention, physical performance tests will be analyzed under both single task and dual-task conditions. Dual-task cost will be calculated to quantify the impact of performing a cognitive task on physical performance. Given the cluster-randomized design, changes over time and between-group differences were analyzed using linear mixed-effects models, accounting for the hierarchical structure of the data, with participants nested within fibromyalgia associations. Group (intervention vs. control), time, and their interaction (group × time) were included as fixed effects. Random intercepts were specified for associations and for participants, and random slopes were included when appropriate, assuming an unstructured covariance matrix. The normality of model residuals was visually assessed for all variables.

To quantify the degree of clustering, the intra-cluster correlation coefficient (ICC) will estimate from the variance components of the mixed-effects models, calculated as the ratio of between-association variance to total variance (between-association plus residual variance). This parameter will be used to evaluate the magnitude of within-association dependence and to confirm the appropriateness of the cluster-adjusted analytical approach.

In the event of participant attrition or missing data due to insufficient attendance, the analyses will follow an intention-to-treat approach, whereby all randomized participants will be included in the analysis according to their originally assigned group regardless of their level of adherence to the intervention. To address missing outcome data, an appropriate imputation strategy will be applied. Where necessary, missing outcome data will be handled using multiple imputations methods. This procedure will be performed following the assumptions for missing at random. When imputation is not feasible, the last observation carried forward or baseline-value carried forward strategies will be applied, depending on data characteristics. In addition, a per-protocol sensitivity analysis will be conducted including only participants who attend the majority of the scheduled sessions to evaluate the robustness of the findings and account for intervention dose–response effects.

The alpha level of significance (0.05) will be adjusted according to the Benjamini–Hochberg procedure to avoid type I error derived from multiple comparisons [55]. Effects sizes were computed and interpreted according to discipline-specific thresholds recommended for physiotherapy and rehabilitation research [56]. For measures of individual differences (Pearson’s r), effect sizes of 0.3, 0.5, and 0.6 were interpreted as small, medium, and large, respectively. For group differences (Cohen’s d or Hedges’ g), effect sizes of 0.1, 0.4, and 0.8 were considered small, medium, and large, respectively.

EEG measures were included as secondary, exploratory outcomes. Although an exploratory hypothesis regarding changes in beta power was formulated based on previous literature [57], EEG analyses were not designed as confirmatory and were conducted to generate mechanistic insights. To reduce the number of comparisons and improve statistical power, regions of interest (ROIs) were defined as a priori by grouping electrodes according to anatomical location: frontal (Fp1, Fp2, F3, F4, and Fz), central (C3, C4, and Cz), and parietal (P3, P4, and Pz). Mean spectral power was computed for each frequency band within each ROI by averaging across electrodes. For exploratory analyses, a 2 × 2 factorial design implemented within the EEGLAB STUDY framework was used to examine EEG data before and after the intervention in alpha and theta bands. Permutation-based statistical testing with 2000 iterations was applied, and multiple comparisons were controlled using the false-discovery rate (FDR) correction.

## 3. Discussion

This study aims to investigate the effects of a six-week physical exercise program combining creative dance and strength training on physical fitness outcomes under both single and dual-task conditions in individuals with FM. It is hypothesized that this intervention will lead to significant improvements in physical performance compared with baseline values and a control group. As a secondary and exploratory objective, the study also aims to investigate exercise-related changes in resting-state brain electrical activity and heart rate variability, with a primary focus on beta power, to explore potential neurophysiological mechanisms underlying functional improvements. It is hypothesized that the intervention may be associated with increased beta power and enhanced autonomic modulation.

Regarding the primary hypothesis, it is expected that the combined strength and creative dance intervention may lead to improvements in agility, muscular power, and mobility after six weeks. A systematic review examining creative or repetitive dance-based training reported enhancements in gait speed, postural control, and balance [58]. Based on this evidence, reductions in completion time are anticipated in the ACE test (gait agility) and the TUG test (functional mobility), while an increase in the number of repetitions performed during the CST under single-task conditions may be observed. In addition, potential improvements in balance performance may be reflected in higher SPPB scores.

These expected physical improvements may be accompanied by a reduction in fear of falling, as assessed by the FES-I, as well as a decrease in fibromyalgia-related symptoms [8], which together could contribute to an overall improvement in quality of life in women with FM. Furthermore, improvements in dual-task performance involving simultaneous physical and cognitive demands may be anticipated [59], reflecting enhanced motor–cognitive integration.

The analyses related to neurophysiological outcomes are exploratory to generate hypotheses regarding potential mechanisms of action. Previous evidence suggests that dance-based interventions may promote neuroplastic changes [10], including increases in gray matter volume in brain regions such as the cingulate cortex, frontal gyri, and limbic system. These regions are closely involved in working memory, executive function, cognitive control, and attentional regulation [10]. Thus, this study may help clarify the consistency and direction of neurophysiological adaptations associated with dance-based exercise, identify potential sources of heterogeneity in previous findings, and generate testable hypotheses regarding the neural mechanisms underlying cognitive and functional changes.

According to the BRAC model, stimulus features, motor responses, and their effects are integrated into so-called “event files” [60], which can later be retrieved to guide behavior. From a neurophysiological perspective, theta activity has been associated with the formation and retrieval of event files, alpha activity with the modulation of these processes, and beta activity with attentional control and the short- and long-term maintenance of event files. Within this framework, creative dance may facilitate the formation, modulation, and maintenance of event files by exposing participants to variable and unpredictable movement situations that resemble real-life balance challenges, particularly when combined with dual-task conditions that are highly relevant to activities of daily living. Hypothetically, this type of intervention may be associated with increased resting-state beta activity and improved autonomic modulation, reflecting a partial normalization of neural and physiological function previously reported in FM [21]. During stimulus–response assessments, additional increases in theta, alpha, and beta activity may be observed, potentially accompanied by improvements in reaction time and response accuracy. Collectively, these adaptations could translate into meaningful functional benefits in everyday life, such as enhanced attentional regulation, executive functioning, and adaptive motor responses in complex environments.

## 4. Strengths and Limitations of This Study

### 4.1. Strengths

This study incorporates a novel multicomponent program integrating strength training and creative dance, simultaneously targeting physical, cognitive, and neurophysiological dimensions of FM. The inclusion of EEG and HRV will provide objective biomarkers to explore brain–body interactions, while the dual-task paradigm offers ecological relevance by addressing real-life motor cognitive demands.

### 4.2. Future Perspectives in Clinical and Research Issues

Future trials should assess (a) longer interventions and include follow-up periods to evaluate sustained effects; (b) measure directly physical activity in both groups; (c) conduct comparative studies contrasting creative versus repetitive dance; (d) include men; and (e) incorporate additional neuroplasticity markers to deepen understanding of underlying mechanisms. If effective, this protocol could support the implementation of dance- and strength-based multicomponent rehabilitation into FM care pathways.

### 4.3. Limitations

This study includes only women, which limits its generalizability to the broader FM population. The intervention period is relatively short, and there is no follow-up to assess the persistence of effects over time, which may not capture long-term adaptations. In addition, the control group will continue with usual care without monitoring physical activity levels, which may potentially attenuate between-group differences. Moreover, the absence of an active control group may introduce placebo effects, which cannot be fully ruled out and should be considered when interpreting the results. Finally, although adequately powered for the primary outcomes, the sample size remains modest for neurophysiological subgroup analyses, particularly the EEG subsample, which may limit the robustness and generalizability of these specific findings.

## Data Availability

The data presented in this study are available on request from the corresponding author due to privacy.

## Supplementary Materials

The following supporting information can be downloaded at https://www.mdpi.com/article/10.3390/sports14020059/s1, Table S1: Music; Document S1: Creative dance training.
