# Supplementary material for: Effects of a Strength and Creative Dance Intervention on Brain Electrical Activity, Heart Rate Variability, and Dual-Task Performance in Women with Fibromyalgia: A Randomized Controlled Trial Protocol
Source: Sports (Basel). 2026 Feb 4;14(2):59. doi: 10.3390/sports14020059 (PMC12944858; doi:10.3390/sports14020059)
Supplement: Supplementary file 1 [file sports-14-00059-s001.zip › Document S1.pdf]

Session 1 BODY

Objective:

- To explore the possibilities of creative movement.
- To expand motor memory
- To develop body awareness
- To enhance spatial awareness (pathways) and temporal awareness (speed/tempo).
- To strengthen self-confidence.
- To learn and apply movement strategies.

Exercise 1

Objective: To encourage the creative exploration of movement and the development of body awareness.
Music: ambient.

Writing with a Body Segment (5 min): Participants, positioned individually and remaining in place, are invited to explore all possible ways of moving each part of their body. At the beginning, the physical educator guides the group through a structured task, such as: *"Write the letter A with your elbow."*. Gradually, participants are given more freedom: *"Write your name or a letter using another part of your body, not the elbow."* They are then encouraged to explore movements of varying amplitude—*large or small gestures*—to expand their expressive range.

Exercise 2

Objective: To learn how to move through space in a controlled manner and to promote the creative exploration of movement in a dynamic way.
Music: ambient.

Trajectories (10 min) Participants move through the space at their own comfortable walking pace, following the lines marked on the floor. During the activity, they must continuously move the body segment indicated by the physical educator at the beginning of the exercise, and later, they may choose freely which body part to move
Variation: When two participants meet face-to-face on the same line, they should continue along their respective pathways, but this time they must avoid each other using a *different movement strategy* than the one used previously.

Exercise 3

Objective: To develop movement awareness in pairs and observational skills (interaction), and to improve motor coordination.
Music: ambient.

Mirror (5 min): Participants work in pairs, facing each other. One participant takes the role of the *mover*, while the other acts as the *mirror*, replicating the movements of their partner. Guided prompts from the physical educator may include: *"Act as if you are getting dressed.", "Imagine you are holding a box; place it in different ways.", "Practice entering a job interview (opening the door, greeting in different ways, deciding where to look).", "Change a light bulb on the ceiling." or "Teach Tai Chi or gym exercises in front of a mirror to observe technique.".* Participants are encouraged to focus on attention, precision, and the quality of movement while observing their partner.

Exercise 4

Objective To learn to move through space and adapt to different walking rhythms and movement patterns.
Music: ambient.

Passing (10 min): participants move through the space, seeking open areas and varying their walking speeds according to the following scale: (0: stop; 1: slow;2: comfortable walk;3: brisk wal;4: fast walk and 5: very fast walk). Initially, the physical educator provides guidance on how to move and which body segments to emphasize, for example: *"on tiptoes, on heels, long strides, lateral steps while moving your arms,"* etc.
Variation: The educator indicates the speed of movement. When participants encounter a partner, they stop for 10 seconds and perform the previous exercise freely, with one participant leading the movement and the other mirroring.
Variation: Same as Variation 1, but the exercise is performed only when participants make eye contact.

Exercise 5

Objective: To facilitate a cool-down and learn individual compensatory stepping strategies.
Music: ambient and directive

Peripheral Start (5 min): The physical educator demonstrates stepping strategies, such as a single step forward or laterally. Participants then experiment individually with different ways of initiating a movement that leads into the taught steps, exploring how each action can begin and end.
Variation: Perform the exercise at different levels (e.g., high, medium, low) to explore spatial and bodily variations.

Session 2 BODY

Objective:

- To develop body awareness and movement perception
- To improve motor coordination and control of static and dynamic body supports.
- To explore creative movement.

Exercise 1

Objective: to develop body awareness and movement perception.
Music: ambient.

Guide Me (5 min): Participants work in pairs, stationary in space. One partner closes their eyes while the other gently guides their body, exploring different possibilities of movement. The participant with eyes closed focuses on the areas being touched and performs the movements they feel.
Variation: The guiding partner touches one, two, or three body segments of their partner, who responds by moving them freely. The goal is to explore the greatest possible range of motion and movement possibilities.
Variation: Guiding while walking: one partner keeps their eyes closed and the other keeps their eyes open, placing one hand on the partner's back and the other on their forehead to guide their movement.

Exercise 2

Objective: To increase heart rate and work on coordination skills.
Music: ambient.

Trajectories (10 min): Participants move through the space while walking along lines marked on the floor. During the activity, they move the body segment indicated by the physical educator at the start, and later may choose freely which segment to move. When participants encounter others on their path, one of the two closes their eyes and must guide the partner in order to avoid collision and continue along the trajectory.

Exercise 3

Objective: To explore different body supports and work on weight shifts.
Música: directive.

Explore Supports (5 min): First, the physical educator demonstrates different types of weight shifts (forward, backward, diagonal, etc.) using various foot positions. Participants then move freely through the space, and whenever they hear a specific word or phrase repeated in the music, they perform a weight shift, ensuring it is different from the previous one.
Variation: The physical educator specifies the direction of the weight shift.

Exercise 4

Objective: To explore and become aware of the support surface while moving, and to improve decision-making speed.
Music: ambient.

Passing (10 min): Participants move individually through the space, seeking open areas and varying their walking speeds according to the following scale: (0: stop; 1: slow;2: comfortable walk;3: brisk wal;4: fast walk and 5: very fast walk). Whenever participants cross paths with a partner, they perform a weight shift. Responses cannot be repeated until at least three movements later.
Variation: If one partner performs a lateral weight shift to the right, the other must respond in the opposite direction or with a different movement. Participants are encouraged to react as quickly as possible.
Variation: Perform the passing activity while moving with maximum amplitude of the body segments.

Exercise 5

Objective: To recall and practice compensatory stepping strategies.
Music: ambient.

Puppet (5 min): The physical educator demonstrates single steps forward and lateral (weighted and unweighted). Participants work in pairs: one as the puppeteer and the other as the puppet. The puppeteer touches a part of the partner's body and imagines one or more strings attached. They pull the strings at different speeds, heights, and directions, while the puppet responds accordingly. Movements should conclude with actions that incorporate compensatory stepping strategies..

**Variation:** Perform the exercise at different levels (e.g., high, medium, low).<br>**Variation**: Colored footprints are placed on the floor (red, blue, and green). Red = left foot, Blue = right foot, Green = lower the center of gravity. Pairs perform the puppet exercise following the footprints, arranged in rows.

**Session 3 WEIGHT**
**Objective:**<br>- To develop postural awareness and control.<br>- To promote adaptation and creativity in motor displacements.<br>- To strengthen group trust and cooperation
**Exercise 1**<br>**Objective:** to explore changes in body weight.<br>**Music:** Ambient.<br><br>**Animals** (5 min): Participants move through the space, embodying the animal named by the physical educator (e.g., elephant, eagle, horse, giraffe, cheetah, turtle, chimpanzee, kangaroo, etc.), exploring different ways of shifting weight and moving.
**Exercise 2**<br>**Objective e:** To improve the ability to vary control and distribution of body weight<br>**Music:** Ambient.<br><br>**Trajectories** (10 min): Participants walk along the lines marked on the floor. When they hear the following numbers, they perform: 1: light steps, 2: heavy steps, 3 very quick steps (minimal contact time) and 4: slow steps.<br>**Summative Variation:** Participants must perform the opposite of what the physical educator indicates, for example: tiptoes (heels), forward (backward), straight (curved), high (low), lateral right (lateral left), etc.
**Exercise 3**<br>**Objective:** To explore body control and trust in a partner.<br>**Music:** Ambient.<br><br>**Trust** (5 min): Participants work in pairs and practice different ways of allowing themselves to fall while their partner absorbs the impact. The exercise progresses from low to high levels: Sitting, falling backward; Squatting, falling to the side and Standing, falling forward.
**Exercise 4**<br>**Objective:** To develop adaptation to body control and weight shifts at different levels.<br>**Music:** Ambient.<br><br>**Passing** (10 min): Participants move through the space, seeking open areas and varying their walking speeds according to the following scale: (0: stop; 1: slow;2: comfortable walk;3: brisk wal;4: fast walk and 5: very fast walk). When participants make eye contact with a partner, one assumes the role of *pendulum-statue* (locking their body) and allows their partner to absorb their weight, then restore them to a balanced standing position so they can continue moving. At the next encounter with another partner, the method of falling or transferring weight must be different. The participant in the *pendulum-statue* role must change their posture each time they assume this role, gradually exploring more challenging situations of imbalance and using both low and high levels.
**Exercise 5**<br>**Objective:** To explore weight shifts on different imagined textures and adapt compensatory stepping strategies according to terrain.<br>**Music:** Ambient.<br><br>**Magical Terrain Challenge** (5 min): The floor is marked with tape to indicate different types of surfaces, such as quicksand, ice, lava, sea, etc. Participants must explore various ways of moving when encountering these zones, adapting their movements to the imagined context. During this activity, emphasis is placed on the use of stepping strategies (single step, lateral, cross, backward, etc.).

Session 4 WEIGHT
**Objective:**<br>- To develop the capacity for body control and weight shifts.<br>- To promote adaptability in body weight changes in dynamic situations at different walking rhythms.
**Exercise 1**
**Objective:** To explore and control weight distribution at different speeds.<br>**Music:** Ambient.<br><br>**Displacements and Rhythms** (5 min): Participants walk through the space using different modes: on tiptoes, on heels, normal walk, forward or backward, strides, lateral, turns, etc. When the physical educator claps once, participants move slowly; two claps = moderate speed; three claps = fast speed.
**Exercise 2**
**Objective:** To develop the ability to adapt and control weight while evading, using fluid and balanced movements.<br>**Music:** Ambient.<br><br>**Trajectories** (5min): Participants walk along the lines marked on the floor. When they encounter a partner, they must evade using previously practiced movement patterns. Verbal communication is not allowed; participants must observe subtle cues to respond or adapt their movements.<br>**Variation:** Evade using compensatory stepping strategies
**Exercise 3**
**Objective:** To explore variations in body weight using different movements and levels.<br>**Music:** Ambient.<br><br>**Stepping on Papers** (10 min): Participants work in pairs. One partner selects a target point and uses sheets of paper (minimum 8–10 per pair) to trace a path. They must move along this path using varied movements, which the partner then imitates. Partners switch roles after completing the path. Types of movements include lateral steps, large strides, cross steps, lunges, turning steps, or changes in height.
**Exercise 4**
**Objective:** To explore control and variation of body weight, adapting to walking rhythm.<br>**Music:** Ambient.<br><br>**Passing** (5 min): Participants move through the space, seeking open areas and varying their walking speeds according to the following scale: 0: stop; 1: slow;2: comfortable walk;3: brisk wal;4: fast walk and 5: very fast walk. When participants cross paths, they mutually decide on the number of movements to perform and specify how each should be executed. For example: 5 movements (1 light step, 2 heavy steps, 1 turn, and 1 low-level step). Participants have 10–15 seconds to plan and perform the sequence.
**Exercise 5**
**Objective:** To explore weight control and balance recovery in simulated imbalance situations.<br>**Music:** Ambient.<br><br>**Tightrope Walker** (5 min): Participants walk along an imaginary or marked line, acting as if it were a tightrope. During the activity, they practice reacting to loss of balance with corrective movements. If they step off the imaginary tightrope, they must use a different stepping strategy than previously employed (single step, lateral, cross, backward, medial, etc.).<br>**Variation**: Mark trajectories on the floor in the shape of a number, letter, or punctuation symbol for participants to follow.<br>**Summative Variation:** he partner must now observe and try to guess the trajectory drawn.

Session 5 CONTACT
**Objective:**<br>- To develop body awareness, movement control, and adaptability<br>- To promote physical connection, coordination, and creative motor exploration through body contact.

Exercise 1

Objective: To promote creativity and connection through physical contact and stillness.
Music: Ambient

Create Your Statue (5 min): Participants work in pairs. One partner moves their body segments freely, while the other touches a body part every 10 seconds to "turn it to stone," gradually creating a statue according to their preference. After completing the statue, partners switch roles.
Variation: In trios, two participants act as the "molders" to create statues

Exercise 2

Objective: To develop body awareness and adapt movement.
Music: Ambient.

Trajectories (5 min): Participants walk along lines marked on the floor, moving whichever body segment they choose. Certain participants, designated by the physical educator, take on the role of "freezer" by touching a joint to immobilize it. The designated participant must continue moving while keeping that joint frozen for 15 seconds.

Exercise 3

Objective: To promote synchronization and awareness of constant physical contact.
Music: Ambient.

Glue (5min): Participants work in pairs arranged in rows and move to the other side of the space. The physical educator indicates which body parts should remain in contact (e.g., elbow to head, arm to torso, head to shoulder). Participants must maintain these contacts while moving.
Variation: Trios instead of pairs.
Variation: Midway through the path, the educator indicates new body parts to maintain contact. Participants must quickly adjust and continue moving
Variation: In pairs, share body weight and shift it toward a common center of gravity (concentric and eccentric work). *Eccentric:* holding hands, shifting weight backward, and in this position, changing level, moving, or dancing.

Exercise 4

Objective: To develop the ability to adapt and control weight in situations of imbalance.
Music: Ambient.

Passing (10 min): Participants move through the space, walking at different speeds: 0: stop; 1: slow;2: comfortable walk;3: brisk wal;4: fast walk and 5: very fast walk. Movements are performed along curved paths. Participants may move anybody segment they choose. Certain participants, designated by the physical educator, take on the role of "freezers," shaping a partner's body into a statue for 10 seconds. The statue may not move until 2 seconds after receiving a hug from a partner. When it moves, it must shift weight in the direction of imbalance to recover equilibrium.

Exercise 5

Objective: To explore and become aware of movement based on the body parts involved.
Music: Ambient.

Movement Engine (5 min): Participants work in pairs. One partner moves a body segment (lower body) indicated by their partner through gentle contacts such as light taps, blowing, or pressing. The moving participant executes locomotion using compensatory stepping strategies, always directing the touched limb. They must perform at least two different strategies during their turn, and after switching roles, perform two additional different strategies.

Session 6 CONTACT

Objective:
- To explore and develop awareness of physical contact and its influence on movement.
- To promote non-verbal communication.
- To strengthen coordination and mutual trust for problem-solving.

Exercise 1

Objective: To explore physical contact through dissonance and balance.
Music: Ambient.

<u>Positive and Opposites</u> (5 min): Phase 1 – Individual: Participants move freely through the space, exploring polarity: e.g., hand and knee as positive or opposite poles. Phase 2 – Pairs: Participants assign different polarities consensually. For example: touch the partner's knee with your hand. If both are positive poles, the partner repels the contact. If one is positive and the other negative, the negative pole moves to break contact while the positive pole seeks it. Participants explore balance, force, and resistance through these interactions.

Exercise 2

Objective: To promote physical interaction and dynamic adaptation to polarity.
Music: Ambient.

<u>Trajectories</u> (5 min): Participants walk along lines marked on the floor. They wear vests indicating polarity (white = positive pole, green = negative pole). The physical educator designates the affected area(s) for polarity. When participants encounter a partner, they must act according to their polarity and find a way to continue along their path without stepping off the line.

Exercise 3

Objective: To improve coordination and spatial connection through visual and physical contact.
Music: Ambient.

<u>Find Me</u> (5 min): Participants move through the space, walking in patterns that trace different geometric shapes (triangle, square, circle, rectangle, spiral, etc.), keeping their gaze focused on the floor. The educator then pauses the activity and asks each participant to choose a partner. Participants continue moving along geometric shapes, but now they must keep their eyes fixed on the right shoulder of their partner.

Exercise o 4

Objective: To promote synchronization and non-verbal communication through physical contact.
Music: Ambient.

<u>Passing</u> (10min): Participants move through the space, seeking open areas and walking at different speeds: 0: stop; 1: slow;2: comfortable walk;3: brisk wal;4: fast walk and 5: very fast walk. Participants attempt to align back-to-back and lower themselves toward the floor as much as possible, then rise each time they encounter a partner. Verbal communication is not allowed, and movement may only begin after making eye contact.
Variation: Change the points of contact: for example, glutes to hands, belly to belly, or left foot to left foot, exploring different ways of connecting.

Exercise 5

Objective: To improve attention and concentration, and to practice compensatory stepping strategies.
Music: Ambient.

<u>Synchronization</u> (5 min): Phase 1 – Individual: Participants create a sequence of four movements containing: a long lateral step, two displacements, a turn, and a step backward. Phase 2 – Partner Work: One participant executes their sequence while the other maintains hand contact on the first participant's left shoulder. Phase 3 – Partner Work: Both participants execute their sequences simultaneously, with one maintaining contact on the partner's head and the other on the hip.
Variation: The physical educator assigns numbers to different stepping strategies and indicates which one to perform.

Session 7 SPACE

Objective

- To promote group interaction and coordination.
- To improve spatial adaptability.
- To develop problem-solving skills.

Exercise 1

Objective: To explore interpersonal space and create adaptations to others' movements.

Music: Ambient

Join In (5 min): The group is divided into two. One group spreads out across the space, forming static shapes (open or closed). The other group walks around observing these static figures and must approach and physically fit themselves into the open spaces within those shapes
Variation: In pairs, participants must continuously occupy the interpersonal space between them through movements involving different body segments. Partners will rotate periodically to encourage new spatial adaptations.

Exercise 2

Objective: To improve spatial orientation and enhance cognitive function.
Music: Ambient

Trajectories (5 min): Participants walk along the lines marked on the floor. The physical educator designates different zones (e.g., cities, even or odd numbers, colors, etc.). When participants hear the name of a zone, they must move toward it. During displacement, they follow the educator's movement cues: lateral steps, backward walking, long strides, tiptoes, heels, turns every three steps, zigzag patterns, etc.
Variation: Move toward the *opposite* zone from the one indicated.

Exercise 3

Objective: To foster creativity in the use of space within a group through movement.
Music: Ambient

Group Figures (5 min): The group is divided into two teams. Each team decides how to begin the activity, following the only rule: they must create shapes or formations, and no one may separate from the group. The teams then move through the space simultaneously, attempting to avoid collisions with each other. As the activity progresses, the available space is gradually reduced to increase difficulty and encourage new problem-solving strategies. Teams may not repeat a formation and must create at least two different shapes before returning to a previous one.
Variation: The educator specifies how the movement should occur (e.g., only moving to the right, moving backward).
Variation: One group moves at a slow pace while the other moves very quickly

Exercise 4

Objective: To develop spatial adaptability and body awareness.
Music: Ambient

Passing (5 min): Participants move through the space at different speeds (0 = stop; 1 = slow; 2 = comfortable walk; 3 = brisk walk; 4 = fast walk; 5 = very fast walk). As they move, they must adapt their body posture and movement to represent the spatial form indicated by the physical educator — for example: a balance beam, a small, medium, or large box, a flat or three-dimensional triangle, or a ball.

Exercise 5

Objective: To improve agility, coordination, and spatial adaptability in order to maintain balance.
Music: Ambient

Dodge (10 min): Participants line up in rows and move in pairs, one pair at a time. The first person in each pair sets the walking rhythm and direction. The partner behind must stay as close as possible. At any moment during the walk, the leader may stop suddenly or shift laterally to the left or right, and the follower must dodge using compensatory stepping strategies (e.g., single step, lateral step, crossover step, medial step, etc.).
Variation: Partners face each other at opposite ends, maximum distance 6 meters (repetition encouraged). One partner calls out an *even* or *odd* number when facing their teammate (even = dodge to the right; odd = dodge to the left). In the next round, they must do the opposite of what was said.
Variation: Same as the previous variation, but now the leader can decide to slightly raise their arm; the follower must respond by dodging, ducking, continuing forward, or adding another movement strategy.

Session 8 SPACE

Objective

- To enhance spatial awareness and flexibility.
- To improve motor and body coordination.
- To stimulate mental agility.

Exercise 1

Objective: To improve flexibility in spatial movement according to a designated focus point.

Music: Ambient

Draw (10 min): The physical educator indicates a shape to be drawn through movement (e.g., hexagon, rectangle, triangle, or a letter). Participants must trace the shape while maintaining their gaze on the focus point indicated by the instructor.
Variation: Groups of three participants perform the shapes according to the indicated focus point.
Variation: The educator specifies the type of movement to be used for the displacement.

Exercise 2

Objective: To improve spatial attention and body control.
Music: Ambient

Trajectories (5 min): Participants walk along the lines marked on the floor. The physical educator instructs them to perform the movement while keeping their gaze fixed on a specific focus point.
Variation: Perform the opposite type of movement from the one indicated by the educator.
Variation: Add obstacles in the space (e.g., cones) to increase spatial awareness and control.

Exercise 3

Objective: To stimulate creativity and improve coordination, as well as spatial and body control.
Music: Ambient.

Create Your Path (10 min): Each participant draws on paper a personal pathway that includes a variety of movements such as straight-line walking, lateral displacements, jumps, zigzags, etc. Once finished, participants spread out across the space. At the cue "Go!", everyone begins to follow their self-designed trajectory.
Variation: After completing the pathway, participants must return to the starting point walking backward.
Variation: The physical educator specifies how to move (e.g., lateral, a turn every four steps, marching speed, medium level, feet together one in front of the other, long strides, etc.).
Variation: Assign an attentional focus point in the space during movement.

Exercise 4

Objective: To improve spatial orientation and motor coordination.
Music: Ambient

Passing (5 min): Phase 1: Participants create a sequence of four movements: two different displacements, a jump, a turn, and a movement at a different level. The educator designates four focus points in the room (e.g., colors or cities). Phase 2: Participants move through the space at different speeds: 0: stop; 1: slow; 2: comfortable walk; 3: brisk walk; 4: fast walk and 5: very fast walk. When the physical educator calls out a focus point, participants perform their four-movement sequence while keeping their gaze directed at that focus.
Summative Variation: In addition to indicating speed, the educator specifies a shape or letter to perform while walking or drawing trajectories on the floor.
Variation: The educator calls a focus, but each movement must be performed toward a different focus point — all to the right or left according to colour order, or freely.

Exercise 5

Objective: To improve spatial perception, agility, and coordination.
Music: Ambient

Advanced Dodge (10 min): Participants work in pairs and move through the space (configuration may vary). One participant acts as the leader while the other follows as closely as possible. The leader changes walking rhythm, direction, or pace without prior notice.
Variations: verbal instructions (jump, duck, left, right
Variation: verbal instructions dodge in a direction indicated by raising the left or right arm.
Variation: Dodge to the opposite side of the cue.
Variation: The leader performs rapid turns or sudden stops combined with movements.

**Session 9 TIME**
**Objective**

- To improve adaptation to rhythm and timing of body movements
- To promote coordination and fluidity in movement.

Exercise 1

Objective: To improve the ability to adapt to variations in rhythm and timing, and to enhance body control.
Music: directive.

Timing (5 min): Participants move through the space, occupying different areas, following the rhythm set by a metronome (APP). The physical educator indicates the number of beats (e.g., 4, 6, 8, 10) within which participants must perform the same movement simultaneously (e.g., squat, lie down, sit, lateral lunge, etc.). Once the time is up, everyone continues moving until the next number of beats is called to perform the movement again.

Exercise 2

Objective: To improve the ability to move in a fluid and controlled manner, and to enhance temporal awareness and body coordination.
Music: Ambient
Trajectories (5 min): Participants move along the lines marked on the floor. They perform the same movements as in the previous activity, but must stay within the designated space and adapt to other participants encountered along the way. The physical educator indicates which body segments to move in time with the metronome.

Exercise 3

Objective: To improve time perception and synchronize body movements.
Music: Ambient.

Move to My Tempo (5 min): Participants work in pairs, distributed throughout the space. One partner takes on the role of setting the tempo by producing vocal sounds.
Variation: Specify a single joint or body segment to move.
Variation: Specify two joints or body segments to move.
Variation: Incorporate compensatory stepping strategies, including the "Stop–Reverse" function, while maintaining tempo set by the metronome (APP).

Exercise 4

Objective: To improve the ability to adapt movements in response to music.
Music: directive.

Passing (5 min): Participants move through the space in time with the music, synchronizing their steps with the musical rhythm or metronome (APP). During the activity, the physical educator gives instructions on what to do, such as types of displacement, right turns, tracing a hexagon, or adjusting the level/intensity of the movements.

Exercise 5

Objective: mejorar la capacidad de adaptación al tiempo y flexibilidad de los movimientos.
Music: Ambient

Stop and Go (10 min)**:** Participants work in pairs, moving through the space. One partner has the role of giving commands: saying "STOP," which signals the other partner to halt, and "GO," which signals them to continue. The command-giver varies the duration of the pauses. During this activity, participants perform a sequence of movements that incorporate compensatory stepping strategies. They explore trunk inclination, base of support, types of displacement, step lengths (short and long), level changes, and other movement resources taught in previous sessions.

Session 10 TIME

Objective

- To improve postural control and time perception.
- To develop adaptations in different contexts.
- To promote creativity and movement fluency.

Exercise 1

Objective: To improve the ability to adapt to variations in rhythm and timing, enhancing body control..
Music: directive (APP)

Frozen Rhythm (5 min): articipants move through the space following the metronome rhythm. When the music stops, everyone must immediately freeze in their current position, maintaining control of their posture until the music resumes. The tempo of the music or metronome can vary from slow to fast, and participants must adjust their movements accordingly.
Variation: When frozen, participants may be instructed to maintain a low-level posture, perform a turn, or assume a geometric shape. The educator can also ask them to hold the position for a specific interval (e.g., 4 beats, 8 beats).

Exercise 2

Objective: To improve the ability to adapt movement to time and rhythm.
Música: Music: directive (APP)

Trajectories: Participants move along the lines marked on the floor. The physical educator indicates the tempo of the displacement (e.g., slow motion, fast, very fast, in time with claps, or contrasting with the music—e.g., moving fast to slow music and vice versa).
Variation: Participants create a short movement phrase consisting of four daily-life actions (e.g., combing hair, chopping onions, dressing, taking a shower). This sequence must be performed while moving along the lines, adapting to the tempo indicated by the educator. Numbers can be assigned to participants to create variability in execution tempos, requiring adaptation. Movements along the lines can be performed in slow motion or fast motion, applying all actions in the phrase.

Exercise 3

Objective: To improve group synchronization and movement adaptability according to tempo.
Music: Ambient

Movement Canon (10 min): Participants are organized in three rows (4 people per row). The first three participants in each row create a movement sequence that repeats until reaching the end. This sequence should include lateral displacements, turns, jumps, steps backward, steps forward, and different levels. If the sequence is too complex, the physical educator provides a movement sequence.
The first three participants start simultaneously; when they finish the sequence and begin again with the first movement, the next three participants in the row start, continuing in canon style.
Variation: Adjust the sequence to the music or tempo indicated by the educator, using claps or a metronome.

Exercise 4

Objective: To improve the ability to adapt movements in response to music.
Music: directive.

Passing (5 min): Participants move through the space in time with the music, synchronizing their steps with the musical rhythm. During the activity, the physical educator provides instructions on what to do, such as types of displacement, right turns, tracing a hexagon, or adjusting the level/intensity of the movements.

Exercise 5

Objective: To improve the ability to adapt to timing and enhance movement flexibility.
Música: none

Percussion (10 min)**:** Participants work in pairs, moving through the space. One partner takes on the role of producing body percussion sounds and varies the duration of the silences. Each time a sound is produced, the other partner performs a sequence of movements incorporating compensatory stepping strategies. Participants explore trunk inclination, base of support, types of displacement, step lengths (short and long), level changes, and other movement resources taught in previous sessions.

Session 11 INTERACTION

Objective

- To promote attention and active listening.
- To improve trust.
- To develop group cooperation and coordination.

Exercise 1

Objective: To improve attention, trust, and non-verbal communication.
Music: Ambient

Follow the leather (5 min): Participants work in pairs and move in rows, progressing to the end of the space using free movement inspired by previous sessions. The partner behind follows, imitating the leader.
Variation: Perform opposite movements, except for walking forward or backward.
Variation: Work in groups of 4–5 participants or the entire group.

Exercise 2

Objective: To promote cooperation and trust between participants.
Music: Ambient

Trajectories (5 min): Participants work in pairs. One partner moves along the lines marked on the floor, while the other provides resistance to their progress by adjusting points of contact (e.g., holding the waist, one hand on the shoulder, palms on the thighs, using the whole body in the direction of movement). Partners then switch roles.

Note: If following the lines is difficult, a predefined trajectory can be established from point A to point B. The trajectories of different pairs may cross, requiring participants to pay attention to others' movements, treating other bodies as obstacles.
Variation: Adjust execution speed (fast, slow, very slow) or spatial levels used (high, medium, low).
Variation: The front partner is blindfolded or closes their eyes. The rear partner guides them along the lines using shoulder gestures. If lines are difficult, participants can move freely in the space, avoiding collisions with other pairs.

Exercise 3

Objective: To explore dynamic interaction between the body and an object.
Music: Ambient

Magnet and Repulsion (5 min) Material: Balloon or ball. Each participant takes a balloon and moves through the space, maintaining contact with the object.
Phase 1: Move the balloon or ball across the body without losing contact (ball negative – body positive).
Phase 2: each time the balloon touches a different part of the body, the participant performs a movement to move away, simulating repulsion (both poles positive or negative)..
Variation: Perform the exercise in pairs, with the premise of always maintaining contact with the object.

Exercise 4

Objective: To foster interpersonal connection through movement.
Music: Ambient

Passing (10 min): The group is first divided into two subgroups. One subgroup is instructed to select a partner from the other subgroup (the second subgroup does not know who has chosen them). Once pairs are established, participants move through the space at different speeds (0–5). During the activity, the physical educator provides instructions. Participants who have chosen partners must approach or move away from their selected partner through different movements, while the group that does not know their partner tries to identify who it is based on the observed movements. Roles are then switched.
Variation: The subgroup that knows their chosen partner is instructed to move closer when their partner moves a right-side body part and move away when their partner moves a left-side body part. The subgroup that does not know their partner is instructed to move different parts of their body freely.

Exercise 5

Objective: To develop communication and cooperation through improvisation.
Music: Ambient

Movement Dialogues (5 min)**:** Participants work in pairs to create a story that generates situations of imbalance, which must be resolved using compensatory stepping strategies. One partner initiates a phrase or word through a motor action, and the other responds, creating an improvised narrative using movements as "words," "phrases," and "punctuation."

Session 12 INTERACTION

Exercise 1

Objective

- To promote cooperation and teamwork.
- To stimulate creativity using objects.
- To improve motor coordination and attention.

**Exercise 1**<br>**Objective:** To promote cooperation, synchronization, and creativity.<br>**Music:** Ambient<br><br>**The Machine** (10 min): The group is divided into two subgroups. Each subgroup must construct a "machine" in which each participant has a specific function. Once the machine is created, participants move together as a coordinated unit.
**Exercise 2**<br>**Objective:** To stimulate creativity and body awareness through the use of objects.<br>**Music:** Ambient<br><br>**Trajectories** (5 min): Participants move through the space at a comfortable walking speed, following lines marked on the floor. Each participant has an object (e.g., stick or cone) and must explore different ways of moving with the object. Examples include using the stick as a cane, as a sword, balanced on the shoulders (like a parade), as an oar, etc.
**Exercise 3**<br>**Objective:** desarrollar la creatividad y la flexibilidad en la interacción con los objetos.<br>**Music:** Ambient<br><br>**Dynamic Mirror at a Distance** (5 min): Participants move through the space in pre-selected pairs, performing predetermined movements. Each participant travels creating geometric shapes, straight lines, curves, and spirals, according to instructions from the physical educator. If one partner performs a squat, the other executes a jump (these actions are agreed upon at the start, and participants must continuously monitor their partner during the activity). Each pair establishes four movements to remember and execute when prompted by either partner.
**Exercise 4**<br>**Objective:** To develop the ability to adapt motor actions according to attentional focus.atencional.<br>**Music:** Ambient<br><br>**Passing** (10 min): Participants move through the space at different speeds indicated by the physical educator (0–5). During the activity, participants must maintain an attentional focus, such as looking at the right wall or the goal at the end of the space. Visual contact with the focus must be maintained while moving. Movements and displacement must adapt according to the attentional focus, which changes periodically. If no objects are present, materials can be placed in the space to serve as focal points.<br>**Variation:** In pairs, participants balance two sticks (or cones) using the palm or a single finger, maintaining pressure without closing the hand, while moving and keeping their gaze on the designated focus.<br>**Variation:** Assign functions to the sticks according to color (e.g., green = low, blue = high).
**Exercise 5**<br>**Objective:** To create situations of imbalance and practice strategies to resolve them.<br>**Music:** Ambient<br><br>**Tell Me a Story** (10 min): The group is divided into two subgroups. Each subgroup creates a story using an object (e.g., fitball, shopping bags, balls, sticks, etc.). Participants work with the object to develop their story. Once the story is ready, it is presented to the other group, who must guess what the story represents. If participants struggle, the physical educator can provide suggestions. Stories should include situations of balance loss, which must be resolved using compensatory stepping strategies.
