# Supplementary material for: Effects of a Strength and Creative Dance Intervention on Brain Electrical Activity, Heart Rate Variability, and Dual-Task Performance in Women with Fibromyalgia: A Randomized Controlled Trial Protocol
Source: Sports (Basel). 2026 Feb 4;14(2):59. doi: 10.3390/sports14020059 (PMC12944858; doi:10.3390/sports14020059)
Supplement: Supplementary file 1 [file sports-14-00059-s001.zip › Table S1.pdf]

Table S1. Music

Song Title	Author(s)	Group	Year	YouTube Link
Atrévete-Te-Te	René Pérez Joglar and Eduardo Cabra Martínez	Calle 13	2006	https://www.youtube.com/watch?v=GUS9KDvlmJk
Chan Chan	Máximo Francisco Repilado Muñoz	BVSC	1984	https://www.youtube.com/watch?v=ha5Y3rMe-NM
Color Esperanza	C. Sorokin, C. López, D. Torres, P. G. Caridad	Diego Torres	2002	https://www.youtube.com/watch?v=3ldnbWFJ1XQ
Centuries	P. Wentz, P. Stump, J. Trohman, A. Hurley, et al.	Fall Out Boy	2014	https://www.youtube.com/watch?v=dfk6i41GCNo
I've Got That Tune	Sly Dee, Zé Matéo, High Ku	Chinese Man	2007	https://www.youtube.com/watch?v=IIVU63vSuf4
Good Times Roll	Grant Kwiecinski, Dominic Lalli	GRiZ	2016	https://www.youtube.com/watch?v=FRSxiiRLQP4
Hit That Jive	Denis Jasarevic	Gramatik	2009	https://www.youtube.com/watch?v=dldqlKqO8oc
Just Jammin'	Denis Jasarevic	Gramatik	2009	https://www.youtube.com/watch?v=5vqXhPxdiC0
Olvídame	Alejandro Guillán	Baiuca	2018	https://www.youtube.com/watch?v=WN8x2GQKf68
Red Sun	Axel Thesleff	Axel Thesleff	2014	https://www.youtube.com/watch?v=i96j5TsNarA
Beggin'	Bob Gaudio, Peggy Farina	Måneskin (Intérprete)	2017	https://www.youtube.com/watch?v=WNYYvHfLCG0
Sinnerman	Nina Simone	N/A	1965	https://www.youtube.com/watch?v=xP7XuE1Fw9s
Makeba	Jain	N/A	2015	https://www.youtube.com/watch?v=59Q_lhgGANc
Can't Stop The Feeling!	Justin Timberlake, Max Martin, Shellback	N/A	2016	https://www.youtube.com/watch?v=ru0K8uYEZWw
Beyond The Veil	Lindsey Stirling	N/A	2014	https://www.youtube.com/watch?v=Qg7L0OQiN78
Mangüeiro	Baiuca	N/A	2019	https://www.youtube.com/watch?v=nyK8UX98_JU
Keep On Keepin' On	Mac Powell	N/A	2024	https://youtu.be/AuG9A3MEuMs?list=RDAuG9A3MEuMs
Lost Girls	Lindsey Stirling	N/A	2016	https://youtu.be/pWn7PYm-W90?list=RDpWn7PYm-W90
Roundtable Rival	Lindsey Stirling	N/A	2014	https://youtu.be/jvipPYFebWc?list=RDjvipPYFebWc
Shatter Me	Lindsey Stirling	N/A	2014	https://youtu.be/49tpIMDy9BE?list=RD49tpIMDy9BE
So U Kno	Tom y Ed Russell	Overmono	2021	https://youtu.be/SRVxRUJxITY?list=RDSRVxRUJxITY
Stay Ft. Mikky Ekko	Rihanna	N/A	2012	https://youtu.be/JF8BRvqGCNs?list=RDJF8BRvqGCNs

Untravel	Rival Consoles	N/A	2018	https://youtu.be/cxxIN6fyj58?list=RDcxxIN6fyj58
Chicken Teriyaki	Rosalia	N/A	2022	https://youtu.be/OG4gq9fCoRE?list=RDOG4gq9fCoRE
Hips Don't Lie	Shakira	N/A	2005	https://youtu.be/DUT5rEU6pqM?list=RDDUT5rEU6pqM
Sia -Elastic Heart	Shia LaBeouf and Maddie Ziegler	N/A	2013	https://youtu.be/KWZGAExj-es?list=RDKWZGAExj-es
The Hygrades	Goddy Oku	Rough Rider	1971	https://youtu.be/AFaqJC_tXP8?list=RDAFaqJC_tXP8
Getaway	The Salsoul Orchestra		1977	https://youtu.be/BlJh43AuSRo?list=RDBlJh43AuSRo
The Anthem	GRiZ	N/A	2015	https://youtu.be/FLgLznrVFcA?list=RDFLgLznrVFcA
Chamegei	Thornato	N/A	2017	https://youtu.be/-nR5XUyt-BA?list=PLcuawrHEGEYo1Mwm8kOEt6954KeF0VyCs
Chapinero	Thornato	N/A	2017	https://youtu.be/hx5G6xOm3Cg?list=RDhx5G6xOm3Cg
África	David Paich and Jeff Porcaro	Toto	1982	https://youtu.be/FTQbiNvZqaY?list=RDFTQbiNvZqaY
Wake Me Up Before You Go-Go	George Michael y Andrew Ridgeley	Wham	1984	https://youtu.be/pIgZ7gMze7A?list=RDpIgZ7gMze7A
Back In Black	Malcolm Young, Angus Young and Dave Evans	ACDC	1980	https://www.youtube.com/watch?v=9vWNauaZAgg
Highway To Hell	Malcolm Young, Angus Young and Dave Evans	ACDC	1979	https://www.youtube.com/watch?v=Cj0KvdbwEIU
Thunderstruck	Malcolm Young, Angus Young and Dave Evans	ACDC	1990	https://www.youtube.com/watch?v=v2AC41dglnM
Ni Tú Ni Nadie	Carlos Berlanga, Nacho Canut and Alaska	Alaska y Dinarama	1984	https://www.youtube.com/watch?v=sH5ROBxWZyw
Bailando	Alaska, Nacho Canut, Carlos Berlanga, Ana Curra y Eduardo Benavente	Alaska y Los Pegamoides	1982	https://www.youtube.com/watch?v=jQyF7IOa0xQ
Bailaré Sobre Tu Tumba	Miguel Costas, Julián Hernández, Alberto Torrado and Germán Coppini	Siniestro Total	1985	https://www.youtube.com/watch?v=RXMg3B1gx10
It's My Life	Bon Jovi, David Bryan, Tico Torres, Hugh McDonald, Phil X y John Shanks and Everett	Bon Jovi	2000	https://www.youtube.com/watch?v=vx2u5uUu3DE

	Bradley			
Cuentame Un Cuento	Jesús Cifuentes<br>Goyo Yeves<br>Alberto García<br>Chuchi Marcos<br>José Sendino<br>Antón Dávila<br>Diego Martín	Celtas Cortos	1991	https://www.youtube.com/watch?v=Tc3QpoeRIBA
¡Chas! - Y Aparezco A Tu Lado	Christina Rosenvinge y Álex de la Nuez	N/A	1987	https://www.youtube.com/watch?v=ydQuHGMsR7k
Cien Gaviotas	Mikel Erentxun, Diego Vasallo and Juan Ramón Viles	Duncan Dhu	1986	https://www.youtube.com/watch?v=2oskr4igPMU
En Algun Lugar	Mikel Erentxun, Diego Vasallo and Juan Ramón Viles	Duncan Dhu	1987	https://www.youtube.com/watch?v=SEOsusFXyuM
La Culpa Fue Del Cha Cha	Jaime Urrutia, Fernando Ferni Presas and Eduardo Edi Clavo	Gabinete Caligari	1990	https://www.youtube.com/watch?v=xlyrZlX02lI
Devuelveme A Mi Chica	David Summers, Rafa Gutiérrez, Javier Molina and Daniel Mezquita	Hombres G	1985	https://www.youtube.com/watch?v=fWkfgEzfhK8
El Ataque De Las Chicas Cocodrilo	David Summers, Rafa Gutiérrez, Javier Molina and Daniel Mezquita	Hombres G	1986	https://www.youtube.com/watch?v=ET8Atukjvzk
Voy A Pasarmelo Bien	David Summers, Rafa Gutiérrez, Javier Molina and Daniel Mezquita	Hombres G	1989	https://www.youtube.com/watch?v=ouhMAT7IX3c
Jardín De Rosas	Mikel Erentxun, Diego Vasallo and Juan Ramón Viles	Duncan Dhu	1986	https://www.youtube.com/watch?v=tmwh2QFq0Q0
Cuando Brille El Sol	Manuel España, Javi Cano, Carlos Muñoz and Paco Villamayor	La Guardia	1990	https://www.youtube.com/watch?v=VNMbN1JNxzY

Lobo Hombre En París	Rafa Sánchez, Mario Martínez, Luis Bolín and Íñigo Zabala	LA UNIÓN	1984	https://www.youtube.com/watch?v=qv6QgO8uyik
Me Duele La Cara De Ser Tan Guapo	N/A	Los Inhumanos	1988	https://www.youtube.com/watch?v=o0hfokUH2AQ
La Bamba	Steve Berlin, David Hidalgo, Conrad Lozano, Louie Pérez César Rosas and Enrique "Bugs" González	Los Lobos	1987	https://www.youtube.com/watch?v=ceZfF84V6UY
Amante Bandido	Miguel Bosé	N/A	1985	https://www.youtube.com/watch?v=DnigcnOxPPc
La Chica De Ayer	Antonio Vega, Nacho García Vega, Carlos Brooking and Ñete	nacha pop	1980	https://www.youtube.com/watch?v=P-nph4Lcd0k
Ni Tú Ni Nadie	Carlos Berlanga, Nacho Canut and Alaska	Alaska y Dinarama	1984	https://www.youtube.com/watch?v=YpJfmIHUojs
No Controles	Vicky Larraz, Marta Sánchez, Sonia Santana, Juan Tarodo, Luis Carlos Esteban, Emilio Estecha, Gustavo Montesano, Marcelo Montesano and Marta Domínguez	Ole Ole	1983	https://www.youtube.com/watch?v=DHIRo-0BA6Y
Voy A Mil	Vicky Larraz, Marta Sánchez, Sonia Santana, Juan Tarodo, Luis Carlos Esteban, Emilio Estecha, Gustavo Montesano, Marcelo Montesano and Marta Domínguez	Ole Ole	1984	https://www.youtube.com/watch?v=7XKsRvB3QNc
Don't Stop Me Now	Freddie Mercury	Queen	1979	https://youtu.be/HgzGwKwLmgM?list=RDHgzGwKwLmgM
Radio Ga Ga	Freddie Mercury	Queen	1984	https://youtu.be/azdwsXLmrHE?list=RDazdwsXLmrHE
Dime Que Me Quieres	Ariel Rot, Alejo Stivel, Julián Infante, Felipe Lipe and Manolo Iglesias	Tequila	1980	https://www.youtube.com/watch?v=X2ET4Ah3mWM

Salta!!!	Ariel Rot, Alejo Stivel, Julián Infante, Felipe Lipe and Manolo Iglesias	Tequila	1981	https://www.youtube.com/watch?v=N9dIl3vEQB4
Embrujada	TINO CASAL	N/A	1983	https://www.youtube.com/watch?v=kNd-kSzYhtQ
Mediterráneo	Joan Manuel Serrat	N/A	1971	https://www.youtube.com/watch?v=7JJByYw9pe0
Vivir Mi Vida	Khaled y RedOne	Marc Anthony	2013	https://www.youtube.com/watch?v=z3dI54JUsjI
La Flaca	Pau Donés	Jarabe de Palo	1996	https://www.youtube.com/watch?v=R2rP8ZU52gU
Clavado En Un Bar	Fernando Olvera	Maná	1997	https://www.youtube.com/watch?v=VR-P413w-us
Bohemian Rhapsody	Freddie Mercury	Queen	1975	https://www.youtube.com/watch?v=jFKBR1ggTMY

N/A: Not Available
